# From EST to structure models for functional inference of APP, BACE1, PSEN1, PSEN2 genes

**DOI:** 10.6026/97320630015760

**Published:** 2019-10-31

**Authors:** Muthusankar Aathi, Shanmughavel Piramanayagam

**Affiliations:** 1 Computational Biology Lab, Department of Bioinformatics, Bharathiar University, Coimbatore-641046, Tamil Nadu, India

**Keywords:** Alzheimer's disease, Curcumin, Hypothetical protein

## Abstract

Successive oxidative stress and biochemical changes results in neuronal death and neuritic plaques growth in Alzheimer's disease (AD). Therefore, it is interest to analyze amyloid-βeta precursor protein (APP), beta-secretase 1 (BACE1), presenilin (PSEN1 and PSEN2) genes from brain tissues to gain insights. Development of potential inhibitors for these targets is of significance. EST sequences of 2898 (APP), 539 (BACE1), 786 (PSEN1) and 314 (PSEN2) genes were analyzed in this study. A contig sequences with APP (contigs 1-4), BACE1 (contigs 5-7), PSEN1 (contigs 8, 9, 10, 11), PSEN2 (contigs 13, 14) except PSEN1 (contigs 10) and PSEN2 (contigs 13) genes were identified. APP (contig 3 without translational error) was further analyzed using molecular modeling and docking to show its binding with curcumin (principal curcuminoid of turmeric) having -7.3 kcal/mol interaction energy for further consideration as a potential inhibitor.

## Background

Alzheimer's disease (AD) is caused due to the structural and functional loss of neurons which shows symptoms like cognitive and memory deterioration, progressive destruction of intellectual activities in day to life and behavioral abnormalities [1]. About 36 million people were found to be affected by AD worldwide in 2010 and it was anticipated to rise 66 million by 2030 and 115 million by 2050 [2]. In India, 3.7 million people were affected by AD [3] and the number of people having AD. Prevalence increases exponentially with age, affecting a little more than 1% in the population aged 65-69 years up to as much as 30-40% in the oldest old [4]. Alzheimer's disease is mainly caused due to the accumulation of β-amyloid peptides [5], which are formed by the action of sequential cleaving of the APP gene which plays an important role in the central nervous system. Proteolytic cleavage of APP by β- and γ-secretase enzymes resulting in the release of neurotoxic Aβ peptides which can aggregate into oligomer is known. A mutation in the APP gene is likely to inhibit α-secretase cleavage which further enables preferential cleavage by β-secretase. Mutations in the PSEN1 and PSEN2 genes (which are components of the γ-secretase complex) results in increased cleavage by γ-secretase at this site. Both these conditions result in the excess production of Aβ peptide. Eventually, subsequent oxidative stress and biochemical changes result in the neuronal death and development of neuritic plaques in AD [6].

Expressed sequence tags are sequenced regions of complementary deoxyribonucleic acid (cDNA) imitates of messenger ribonucleic acid (mRNA) that are expressed in different states and represents element of the transcribed portion of the genome. The EST sequence information plays a vital role in human biology and disease, such as neurological disorders [7]. This helps to identify the functional genes expressed in diseased condition. Mutations in the alzheimer's susceptibility genes APP, BACE1, PSEN1 and PSEN2 greatly increase the risk of AD. The approved drugs for AD namely, tacrine, donepezil, rivastigmine and galantamine failed due to severe side effects and were abandoned. This work will helpto identify the functional annotation of APP, BACE1, PSEN1, PSEN2 genes and new discovery for the development of novel therapeutic approaches for the treatment of AD.

## Methodology

### Retrieval of ESTs sequence and assembly:

In silico analysis of AD human genes APP, BACE1, PSEN1 and PSEN2 taken from UniGene database and those genes originating from brain tissues were taken. The 5' ESTs were considered, as the ESTs generated from the 3' end are most error prone as of the low base-call quality at the start of sequence reads. The 5' EST sequences were extracted using contig assembly program by CAP3 server [8]. The default parameters were used and each gene sequences were submitted to DNA sequence assembly program (CAP3) server in FASTA formatted text file and result was displayed in different output files e.g. contigs, single sequences, Assembly details and sequence file. We have selected contig sequence data set as it is useful functionality ascertained.

### Conceptual translation of ESTs and functional annotation:

ESTScan is a program that can identify the coding regions in DNA sequences and this was translated into amino acid sequences at either N- or C-terminus. Each contig sequence was generated by ESTScan2 tool [10]. Finally, the amino acid sequences were selected using multiple sequence alignment by CLC Genomics Workbench and further functional annotations were carried out. Our translated protein sequences for each sequence were generated by InterProScan 5.0 [11].

### Molecular modelling of hypothetical protein:

Structural annotation of APP hypothetical amino acid sequence was used for build a 3D structure by Modeller v9.13 software [12]. The hypothetical protein sequence was aligned in BLASTP against the Protein Data Bank (PDB) database to select their appropriate templates. The template was selected for hypothetical protein query sequence aligning 18-199 amino acid residues, showing 97% sequence identity with 3KTM [13]; aligning 342-551 amino acid residues shows 99% sequence identity with 3NYL [14] and aligning 652-751 amino acid residues shows 100% sequence identify with 2LP1 [15]. 

These templates were used to build a 3D structure for homology modelling. Modelled structure was energy minimized using Swiss-PDB viewer program (Gromos96 force field). Theoretically predicted structure was visualized using PyMol visualization software. The amino acid constraint validation of the modeled APP protein was done by PROCHECK program (www.ebi.ac.uk/ thornton-srv/software/PROCHECK/) [16]. Further, 3D profile of the modelled protein was computed by Verify3D program.

### Selection of ligands:

The 2D structure of synthetic compounds tacrine, donepezil, rivastigmine, galantamine and natural remedy like compounds from plants such as Rosmarinus officinalis (α-Pinene, Camphene, β-Pinene, 1,8-Cineole, α-Thujone, β-Thujone, Chrysanthenone, Camphor, (+)-Borneol, Bornyl acetate, α-Copaene, Trans-Caryophyllene, α-Humulene, Germacrene-D and (+) -δ-Cadinene); Ginkgo biloba (Quercetin, Kaempferol, Isorhamnetin, Ginkgolide A, B, C, J, M); Panax ginseng (Ginsenoside Rb1 and Rg1); Curcuma Longa (Curcumin, Demethoxycurcumin and Bisdemethoxycurcumin); Salvia officinalis (Borneol, Caryophyllene, Linalool); Huperzia serrata (Huperzine A, B and Lycopodine) ; Melissa officinalis (1-Octen-3-ol, 6-Methyl-5-hepten-2-one, Myrcene, (Z)-β-Ocimene, (E)-β-Ocimene, n-Nonanal, Cis-Rose oxide, (+)-Trans-Rose oxide, (+)-Trans-Limonene oxide, Citronellal, Menthol, Isomenthol, Nerol, Neral, Piperitone, Geraniol, Geranial, α-Cubebene, Geranyl acetate, β-Cubebene, β-Caryophyllene, Valencene, Caryophyllene oxide, 1-Hexadecene, n-Eicosane, n-Heneicosane); Withania somnifera (Propane,1,1-diethoxy-2-methyl-, 2-Nonanone, PhenylethylAlcohol, Amyl nitrite, Dodecanoic acid, 3-ter-Butyl-4-hydroxyanisole, Tetradecanoic acid, n-Hexadecanoic acid, 9-Octadecenal, 1-tridecyne, Oleic acid); Baccopa monnieri (2-octanol, Dimethoxane, 2-Methyl-1-Phenyl-1-butanol, Phytol, Phytol acetate, Octadecanamide); Centella asiatica (Thujopsene, α-Thujene, Eucalyptol, 3-Nonen-2-one, β-Linalool, L-Camphor, trans-Borneol, α–Terpeneol, Cis-Geraniol, Isobornyl acetate, 7-Tetradecene, β-Elemene, β-Gurjunene, γ-Elemene, Isocaryophyllene, Aromadendrene, β-Farnesene, β-Acoradiene, β-Selinene, α-Selinene, α-Chamigrene, α-Panasinsen, -(-)Spathulenol, Viridiflorol, Valeranone, Isoaromadendrene epoxide, Aristolene epoxide, 1-Naphthalenol); Celastrus paniculatus (Palmitic acid, Erucic acid, γ-Muurolene, Cubenol) were downloaded from PubChem databases as . sdf format. Further, the .sdf format converted into .pdb format using Openbabel 2.3.2.

### Molecular docking:

Docking studies was carried out using Glide module from Schrodinger suite [17] to find the interaction between modeled APP protein with natural and synthetic compounds. All the compounds were prepared by LigPrep Module. The protein grids were prepared with the mutated residues and the size of the bounding box was set to 30Å. Modelled APP protein coordinates file of enclosing box was set as x=3.9023Å; y=32.884Å; z=30Å respectively. All the prepared inhibitory compounds were docked against the grid generated APP modelled protein. The inhibitory compounds used for docking was screened using Virtual screening. Glide score was selected as the scoring function to rank the poses of each inhibitory compound. Validation of the docking is useful technique to identify best docked complex among number of docked complex.

## Results and Discussion:

### Retrieval of ESTs sequence:

The EST sequences for human AD genes APP, BACE1, PSEN1 and PSEN2 were searched from UniGene database. The gene entries with their mRNA and ESTs information are listed in Table 1 ESTs of four gene entries originating from brain tissue were used for further analysis.

### EST clustering and assembly:

Each gene sequence of ESTs from brain tissue was retrieved. The 5' ESTs were analyzed, as the ESTs created from the 3' end are most error prone because of the low base-call quality at the start of sequence reads. The subjected ESTs along with their resulting contigs found a total of 988 ESTs from four reported gene entries as listed in Table 5 (Supplementary Material Available in the PDF version). The tissue-based ESTs from four reported genes were subjected to cluster analysis by CAP3 Server. 14 contigs of four genes were found and further analysis was under taken.

### Database similarity searches:

**Figure 1:**
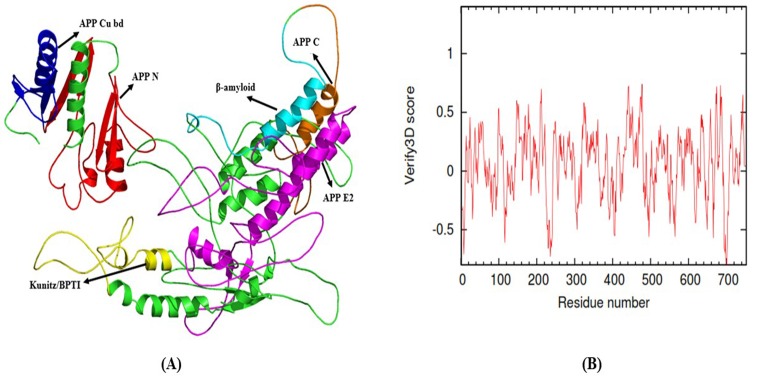
Graphical representation of contig protein sequences obtained from ESTscan2 translation sequences. Red color box represents X error translate level in APP (Contig 1, 2, 4), BACE1 (5, 6, 7), PSEN1 (8,9,11) and PSEN2 (14) except APP contig 3 sequence.

The database similarity search by querying these contigs in BLAT against human genome revealed that alzheimer's contig of APP shows good matches with chromosomes 21. The BACE1, PSEN1 and PSEN2 contigs were showing good matches with chromosomes 11, 14 and 1 respectively and are shown in Table 2. The conceptual translation of 14 contigs sequences in ESTScan2 provides 12 protein sequences from APP, BACE1, PSEN1 and PSEN2, as presented in this analysis and protein sequences were not available for the rest of two contig nucleotide sequences contig 10 and contig 13. Multiple sequence alignment was done for these 12 protein sequences obtained by ESTScan2 tool. The entire alignment shows contig 3 sequence of APP protein alone with no error at translate level and rest of the 11 protein sequences were left due to some erroneous readings (X, which does not code for somewhat amino acids or refers to a stop codon) in their sequence as shown in Figure 1, obtained by CLC Genomics Workbench 7.6. The APP protein sequence of contig 3 is 751 amino acids with a molecular weight of 84818.77 Daltons and this sequence was named as hypothetical protein for further annotation.

### Conceptual translation of ESTs

The APP protein sequence was reported from 5' ESTs of brain tissues and it belongs to the APP amyloid and beta-APP families of proteins with a distinct N-terminal and C-terminal. The major part of the amyloid plaques found in the brains of AD and peptide regions of 36-43 amino acids are fatefully involved in amyloid precursor protein. Aβ molecules can aggregate to form oligomers and the resulting amyloid plaques are toxic to nerve cells [18]. N-terminal region of the APP is a member of the heparin-binding class of GFLDs (Growth Factor-Like Domain) and may itself have growth factor function, neuronal development. It contains four structurally similar domains represented by PFAM families PF12925 [14],PF02177 [19], PF12924 [20] and PF00014 [21] as shown in Table 3. In structural classification by CATH, the classification lineage of hierarchy 3.90.570.10, 3.30.1490.140, 4.10.230.10 is amyloid beta A4 protein; 1.10.287.510 is amyloid protein and 4.10.410.10 is protease inhibitor IX.

### Molecular modelling of hypothetical protein:

**Figure 2:**
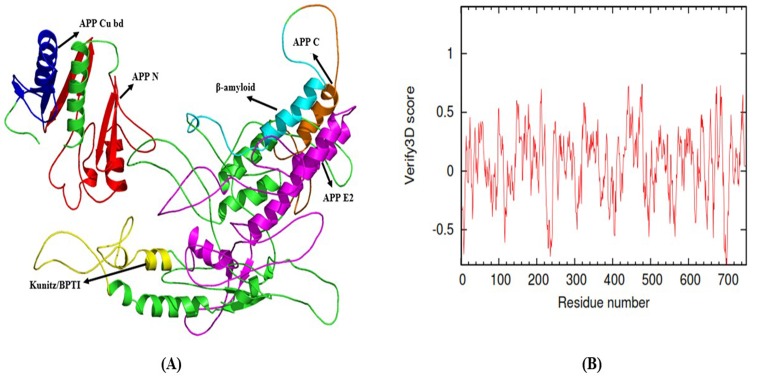
Graphical representation of modelled and validated hypothetical protein of APP. A) Domain regions of red color show APP N (Amyloid A4 N-terminal heparin binding); Blue color is APP Cu bd (Copper-binding of amyloid precursor, CuB); Yellow color is Kunitz BPTI (Kunitz/Bovine pancreatic trypsin inhibitor); Magenta color is APP E2 (E2 domain of amyloid precursor protein); Cyan color is β-amyloid and Orange color is APP C (APP-amyloid). B) Verify 3D plot showed score ranges in between -1.0 to 0.7.

The 3D structure of hypothetical protein of human APP was predicted using MODELLER v9.13. This program was generated ten different 3D modeled structures and validating these structures was considered based on the scoring percentage of the favored regions. Finally, we selected the best modeled structure for hypothetical protein (model 3) as depicted in Figure 2A. Validation of Ramachandran plot showed >96% of the residues in most favored and additional allowed regions and the structure of our modeled protein was found to be stable. Verify3D methods evaluate protein structure using 3D profiles and this program analyzed the compatibility of an atomic model (3D) with their possess amino acid sequence (1D). Each residue is allocated a structural class based on the scores ranges from -1 to +1. In our results verify3D score value of modeled APP protein is -1.0 to 0.7 (Figure 2B). Validation results showed stero chemical properties and geometrical arrangements of the atoms of the protein was stable. The root-mean-square deviation value of modeled APP protein 3D structure was higher (0.439Å) than the existing crystal structure PDB IDs: 3KTM (2.70Å) and 3NYL (2.80Å) with an energy value of -30227.773KJ/mol.

### Molecular docking:

Molecular docking studies were performed for modeled complete sequences of APP protein with current drugs and medicinal compounds. Various synthetic drugs are available against AD such as tacrine, donepezil, rivastigmine and galantamine, but causing side effects like diarrhea, nausea, vomiting etc [22]. Hence, a new drug development is important to cure AD without these side effects. In our study, we have selected 11 medicinal plants such as R. officinalis (α-Pinene, Camphene, β-Pinene, 1,8-Cineole, α-Thujone, β-Thujone, Chrysanthenone, Camphor, (+)-Borneol, Bornyl acetate, α-Copaene, Trans-Caryophyllene, α-Humulene, Germacrene-D and (+)-δ-Cadinene) plant essential oils have a potent effect in patients with symptoms of AD [23] and mentioned 15 natural compounds were identified in this plant using GC-MS analysis. G.biloba extract from leaves has been found to improve the symptoms of AD [24] and this plant compounds like Quercetin, Kaempferol, Isorhamnetin, Ginkgolide A, B, C, J, M. P.ginseng plant extract from root has a potential role in the treatment AD29 and compounds like Ginsenoside Rb1 and Rg1. C.Longa Linn plant extract from root have been used to treat of AD and compounds like Curcumin, Demethoxycurcumin and Bisdemethoxycurcumin [25]. S.officinalis extract from leaf has been found a significant benefit in cognition to the patients with mild to moderate AD and compounds like Borneol, Caryophyllene, Linalool. H. serrata has been studied extensively for it is role in treating AD and this plant leaves had been extracted to identify compounds like Huperzine A, B and Lycopodine. The essential oil is obtained from leaves of M.officinalis compounds 1-Octen-3-ol, 6-Methyl-5-hepten-2-one, Myrcene, (Z)-β-Ocimene, (E)-β-Ocimene, n-Nonanal, Cis-Rose oxide, (+)-Trans-Rose oxide, (+)-Trans-Limonene oxide, Citronellal, Menthol, Isomenthol, Nerol, Neral, Piperitone, Geraniol, Geranial, α-Cubebene, Geranyl acetate, β-Cubebene, β-Caryophyllene, Valencene, Caryophyllene oxide, 1-Hexadecene, n-Eicosane, n-Heneicosane [26] and this plant has been modulate mood and cognitive performance in AD. Compounds like Propane,1,1-diethoxy-2-methyl-, 2-Nonanone, PhenylethylAlcohol, Amyl nitrite, Dodecanoic acid, 3-ter-Butyl-4-hydroxyanisole, Tetradecanoic acid, n-Hexadecanoic acid, 9-Octadecenal, 1-tridecyne, Oleic acid extracted from W.somnifera root [27] are mainly used to treat AD. B.monnieri leaf extract has been used to promote memory increasing activity and treat psycho neurological disorders. GC-MS analysis of this plant identified compounds such as 2-octanol, Dimethoxane, 2-Methyl-1-Phenyl-1-butanol, Phytol, Phytol acetate, Octadecanamide [28]. C.asiatica plant essential oil extract from leaves and GC-MS analysis compounds like Thujopsene, α-Thujene, Eucalyptol, 3-Nonen-2-one, β-Linalool, L-Camphor, trans-Borneol, α–Terpeneol, Cis-Geraniol, Isobornyl acetate, 7-Tetradecene, β-Elemene, β-Gurjunene, γ-Elemene, Isocaryophyllene, Aromadendrene, β-Farnesene, β-Acoradiene, β-Selinene, α-Selinene, α-Chamigrene, α-Panasinsen, -(-)Spathulenol, Viridiflorol, Valeranone, Isoaromadendrene epoxide, Aristolene epoxide, 1-Naphthalenol. This plant has ability to prevent cognitive deficits treatment for AD. C.paniculatus plant contains essential oil extract from seeds and GC-MS analysis compounds like Palmitic acid, Erucic acid, γ-Muurolene, Cubenol. The seed oil is studied as best nervine tonic and used in treatment of various neurological disorders [29]. We validated the efficacy of synthetic and medicinal plants based compounds with modeled APP protein using molecular docking approach to identify the best inhibitor for AD.

**Figure 3:**
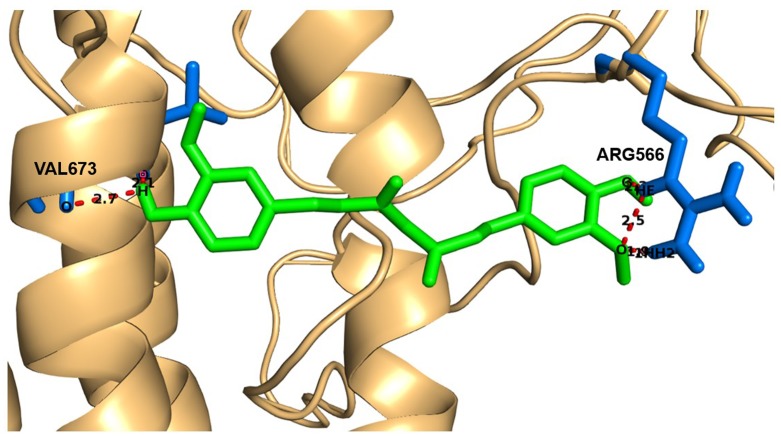
Interaction of modeled protein APP docked with curcumin. Light orange color represents protein; green color is curcumin compound and blue color is interacted residues.

APP is a transmembrane protein without known function that is constitutively cleaved into peptides during cell metabolism. The amyloidogenic 40 or 42 amino acid Aβ peptide is released after cleavage by β-secretase and γ-secretase. Familial alzheimer's disease (FAD) mutations have been identified in APP, PSEN1 and PSEN2 genes, which are essential for the generation of A β peptides [30]. Reported APP mutation sequences include A673V [31], V717I [32]. Figure 3 shows the interaction of modeled APP protein with curcumin having least glide score value of -7.3Kcal/mol and more number of hydrogen bonds (ARG566, VAL673) were formed than other compounds. From the results of docking study, out of 11 medicinal plant compounds only six medicinal plants such as P. ginseng (Ginsenoside Rb1), C. longa Linn (Curcumin), C. asiatica (Aristolene epoxide, Valeranone), B. monnieri (Phytol acetate), B. monnieri (Dimethoxane), C. paniculatus (Erucic acid) and synthetic (Rivastigmine, Tacrine, Galantamine) compounds showed proper interaction but mutated residues docked with ginsenoside rb1 and curcumin compounds (Table 4). Tang and Taghibiglou 2017 [33] has reported curcumin compound to be more effective than current treatment of AD. Alcigir et al. [34] found that positive results in new-born rodent pups, curcumin compound as a natural therapy for permanent treatment based on neuronal impairment. Abdolahi et al. [35] has considered curcumin compound as a novel promising therapy in migraine prevention. From the molecular interaction study, we conclude that, natural compound curcumin shows better interaction than synthetic, other natural screened compounds and AD approved drugs. Hence we suggested as an alternative lead compound of curcumin in alzheimer's disease research.

## Conclusion

EST analysis of the four genes associated with AD produced 14 contig sequences. APP contig 3, the only contig with no error of translation was annotated using functional and structural data. APP was further analyzed using molecular modeling and docking with natural compound of curcumin, it shows the best glide score of -7.3kcal/mol into mutated residues unlike the synthetic and other natural compounds. Hence to avoid the side effects of synthetic drugs and natural compound, curcumin is suggested for the treatment of AD.

## Figures and Tables

**Table 1:** UniGene information on human Alzheimer's disease

S.No	Name of the Genes	Source	mRNA	ESTs
1.	APP	Homo sapiens	38	2898
2.	BACE1	Homo sapiens	27	539
3.	PSEN1	Homo sapiens	16	786
4.	PSEN2	Homo sapiens	10	314

**Table 2:** BLAT output showing the alignment of APP, BACE1, PSEN1 and PSEN2 contigs sorted by score

Query	Score	Start	End	Qsize	Identity (%)	Chromosome	Strand
					APP		
Contig1	547	30	582	583	99.9	21	-
Contig1	515	31	550	583	99.9	21	+
Contig2	734	6	752	780	99.5	21	-
Contig2	734	7	757	780	99.2	21	+
Contig3	3838	1	3876	4579	99.8	21	-
Contig3	3838	1	3876	4579	99.8	21	+
Contig4	1331	2	1340	1340	100	21	+
Contig4	1330	1	1340	1340	100	21	-
BACE1							
Contig5	1605	3	1616	1616	99.9	11	+
Contig5	1604	2	1616	1616	99.8	11	-
Contig6	4916	1	5092	5184	99.4	11	-
Contig6	4916	1	5100	5184	99.3	11	+
Contig7	563	1	572	572	99.7	11	-
Contig7	563	1	572	572	99.7	11	+
PSEN1							
Contig8	546	1	589	589	99.7	14	-
Contig8	545	2	589	589	99.7	14	+
Contig9	4161	1	4265	4265	99.6	14	+
Contig9	4159	1	4265	4265	99.6	14	-
Contig10	465	1	477	604	98.8	14	-
Contig10	464	1	478	604	98.6	14	+
Contig11	1491	1	1501	1680	100	14	-
Contig11	1491	1	1505	1680	99.9	14	+
Contig12	501	44	638	638	99.5	14	+
Contig12	499	38	638	638	98.7	14	-
PSEN2							
Contig13	577	1	580	580	99.9	1	-
Contig13	577	1	580	580	99.9	1	+
Contig14	1499	1	1602	1918	98.4	1	-
Contig14	1467	1	1603	1918	97.9	1	+

**Table 3:** The InterProScan annotations for Hypothetical protein

Protein	GENE3D	PANTHER	PFAM	PRINTS	PROFILE	SMART	SUPER FAMILY
Contig3	G3DSA:	PTHR23103	PF12925;	PR00204	PS00319; 00280;	SM000006; 000131	SF10984;
/APP	3.90.570.10;		PF02177;		320		56491;
	1.10.287.510;		PF12924				89811;
	4.10.410.10		and				57362
			PF00014				

**Table 4:** Molecular docking analysis of modeled APP protein with synthetic and medicinal compounds

S.No	Compound Name	Glide Score (Kcal/mol)	No.of Hydrogen Bonds	Interacting Residues	Ligand Atom	Distance Length (Å)
1.	Curcumin	-8.7	5	ARG566:HE	O	2.3
				ARG566:HE	O	2.5
				ARG566: 1HH2	O	1.9
				VAL673:O	H	2.7
				VAL673:O	H	2.1
2.	Ginsenoside	-6.1	2	VAL673:O	H	1.9
	Rb1			LYS680:H	O	1.9
3.	Aristolene	-5.2	2	PHE745:O	H	2.41
	epoxide			GLU747:O	H	2.21
4.	Phytol acetate	-3.8	1	SER711:O	H	1.98
5.	Dimethoxane	-3.6	1	GLY677:O	H	2.01
6.	Valeranone	-3.6	1	LYS680:O	H	1.69
7.	Erucic acid	-3.5	2	LYS680:H	O	2.37
				ASP720:O	H	1.8
8.	Rivastigmine	-2.9	1	LYS600:O	H	1.99
9.	Tacrine	-1.4	1	ASP720:O	H	1.66
10	Galantamine	-1	1	ASP720:O	H	1.51
11	Donepezil	-	-	-	-	-
